# Effect of Alkali Activation on Swelling Suppression and Microstructural Development in Geopolymer-Stabilized Bentonite

**DOI:** 10.3390/polym18050606

**Published:** 2026-02-28

**Authors:** Tengshen Jing, Shengyang Yuan, Xianfeng Liu, Yulin Liu, Haibin Xu, Weixing Zhou, Pengjie Lin, Guanlu Jiang

**Affiliations:** 1 Key Laboratory of High-Speed Railway Engineering, Southwest Jiaotong University, Ministry of Education, Chengdu 610031, China; 2 School of Civil Engineering, Southwest Jiaotong University, Chengdu 610031, China; 3 School of Civil Engineering, Xinjiang Institute of Engineering, Urumqi 830023, China

**Keywords:** geopolymer, expansive clay, stabilization, swelling characteristics, microstructure

## Abstract

Geopolymers, a class of alkali-activated aluminosilicate binders, have emerged as a sustainable alternative for expansive soil stabilization. In this study, the swelling behavior of geopolymer-treated bentonite was systematically investigated using a Taguchi orthogonal design, complemented by XRD, FTIR, and SEM analyses to elucidate the underlying mechanisms. Specimens were compacted to an initial void ratio of *e* = 1.1, sealed, and cured under controlled conditions (22 ± 2 °C and 70 ± 2% relative humidity) prior to testing. The free swell ratio (FSR) was determined using a standardized free swelling test in accordance with GB/T 50123-2019, which is technically consistent with ISO 17892-13, under zero vertical surcharge. Each orthogonal condition was tested using a single specimen, and the reported values represent individual measurements. The results show that NaOH concentration is the dominant factor controlling swelling response, with a quantified contribution of 55.04%. The swelling behavior exhibits a distinct two-stage trend, characterized by an initial enhancement at low alkali concentrations followed by a significant suppression beyond a critical threshold of approximately 3 mol/dm^3^. Microstructural analyses reveal that this transition is governed by a progressive interlayer cation exchange, the structural dissolution of clay minerals, and the formation of geopolymer gel, which densifies the soil matrix and restricts interlayer expansion. These findings provide quantitative and mechanistic insight into the role of alkali activation in expansive clay stabilization and establish a practical concentration threshold for optimizing swelling suppression.

## 1. Introduction

Expansive soil is widely distributed around the world, with a significant volume during wetting and drying cycles [1,2]. This cyclic swelling and shrinkage induces cracks, reduces soil integrity, and weakens strength [3], which affects the serviceability of overlying infrastructures [4,5]. Field surveys show that over 60% of new projects in expansive soil areas experience cracking from soil expansion, and about 10% suffer serious, often irreparable damage [6]. Improving the mechanical behavior of expansive soils is therefore critical for infrastructure durability [7]. Remediation techniques generally include pile reinforcement [8], soil replacement [9], and soil stabilization [10,11,12,13].

Soil stabilization methods can be further classified into three categories: physical, biological, and chemical approaches. Physical stabilization improves the strength and gradation of expansive soils by incorporating materials such as sand [14], silt [15], or gravel [16]. It offers simple construction, minimal environmental impact, and avoids chemical additives, but its effectiveness is limited in deep or highly expansive strata. Biological stabilization employs microbial activity to alter pore structures or precipitate cementing agents within soils [17,18]. It is environmentally sustainable but acts slowly, depends heavily on climatic and soil conditions, and often fails to achieve the strength levels required for heavy-load applications [19]. Chemical stabilization modifies the soil’s mineralogy and microstructure by introducing reactive agents such as lime [20], cement [21], or fly ash [22]. These reactants react with clay minerals, primarily montmorillonite, to disrupt swelling lattices or generate cementitious bonds that bind soil particles. It reduces swell potential and enhance strength, providing long-term durability and broad applicability in transportation infrastructures and building construction [9,12,23]. For instance, cement hydration produces compounds that fill soil pores and enhance strength [24]. Nonetheless, long-term performance may decline due to cracking of the cementitious matrix. Moreover, cement and lime production are energy-intensive and emit significant CO_2_ and SO_2_, accounting for roughly 7–8% of global CO_2_ emissions [25,26].

Geopolymers, one type of alkali-activated aluminosilicate binder synthesized from natural minerals or industrial byproducts, were gradually introduced into industry as a sustainable alternative for cement and lime by Davidovits [27]. They react with soil minerals to form stable, low-permeability composites while maintaining the natural soil fabric, and show a good performance in strength, durability, and chemical resistance [28]. Geopolymers typically use aluminosilicate precursors such as fly ash, slag, or metakaolin [28], where fly ash, a byproduct of coal combustion, is abundant but costly to dispose of, making it an attractive, sustainable precursor [29]. They have been successfully applied to the improvement of soft [29,30], saline [31,32], and clayey soils [26,33], though research on expansive soils remains limited [34], especially for the swelling behavior of modified expansive clay. Zhang [35], for example, found that metakaolin-based geopolymers yielded a lower free swell ratio (FSR) than lime-treated sulfate soils, attributing this to the absence of ettringite formation commonly seen in lime-stabilized specimens. However, the modification mechanism of geopolymer-stabilized expansive clay is not clearly demonstrated from microstructural aspects.

Despite the increasing use of fly ash-based geopolymers for clay stabilization, their effectiveness in controlling the swelling behavior of expansive bentonite remains insufficiently understood. Previous studies showed that alkali-activated binders improve mechanical properties and reduce expansion potential, primarily focusing on strength rather than swelling evolution [3,25,33]. However, swelling behavior under varying alkali activation conditions has not been systematically evaluated, and the relative importance of key factors remains unclear. Moreover, the competing mechanisms governing swelling evolution, including cation exchange, mineral dissolution, and geopolymer gel formation, have not been clearly distinguished. Therefore, a systematic framework integrating quantitative parameter analysis and microstructural characterization is necessary to clarify these mechanisms and optimize the geopolymer stabilization of expansive bentonite.

This study conducted an exploratory investigation into the effectiveness and underlying mechanisms of using geopolymers to modify the swelling properties of bentonite. Free swelling tests were carried out to examine the effects of alkali activator concentration, fly ash content, and moisture content on its swelling behavior. Furthermore, SEM, XRD, and FTIR analyses were employed to elucidate the mechanisms governing these effects. The findings aimed to provide insight into understanding and optimizing the use of geopolymers in mitigating the swelling characteristics of bentonite.

## 2. Materials and Testing Program

### 2.1. Materials

The commercial bentonite (Ningcheng Xiangxi Chemical Co., Ltd., Chifeng, China) used in this study had a liquid limit of 136.33%, a plastic limit of 52.15%, a specific gravity of 2.50, and a free swell ratio (FSR) of 102.5%. According to the particle size analysis (Figure S1, Supplementary Materials), the material is predominantly composed of silt- and clay-sized particles, with more than 95% of the grains finer than 0.02 mm, which is consistent with the expected gradation of high-plasticity bentonite. Mineralogical analysis indicates that the bentonite is primarily composed of calcium montmorillonite, with minor amounts of quartz, cristobalite, feldspar, and calcite.

Commercial fly ash (Borun Casting Materials Co., Ltd., Zhengzhou, China) was used as the aluminosilicate precursor for alkali activation. The material was commercially sourced and appears as a gray ultrafine powder with a typical median particle size (D_50_) of approximately 6 μm, reflecting its high fineness and potential reactivity. Based on its oxide composition (SiO_2_ 45.1%, Al_2_O_3_ 24.2%, and CaO 5.6%) and relatively low calcium content, the material is classified as Class F fly ash according to ASTM C618 [36]. The mineralogical composition and oxide constituents were characterized using X-ray diffraction (XRD). The diffraction pattern (Figure S2, Supplementary Materials) indicates that quartz and mullite are the dominant crystalline phases, with minor contributions from calcium oxide and corundum. The reported oxide composition values were obtained from phase identification and semi-quantitative analysis of the XRD results. The fly ash exhibits a liquid limit of 31.77%, a plastic limit of 18.48%, and a specific gravity of 2.30.

Sodium hydroxide (NaOH, purity > 98.5%, Tianjin Botian Chemical Co., Ltd., Tianjin, China) was employed as the alkaline activator. NaOH was selected because it is a widely used hydroxide activator for fly ash-based alkali-activated systems and provides both OH^−^ ions to promote aluminosilicate dissolution and Na^+^ ions to balance charges during gel formation and participate in interlayer cation exchange with montmorillonite. Alternative activators (e.g., KOH, sodium silicate, or blended alkaline solutions) may alter dissolution kinetics, gel chemistry, and ionic strength, and could therefore shift the swelling response and the critical concentration threshold.

### 2.2. Testing Programs

Because geopolymerization involves multiple interacting variables—namely fly ash content, NaOH concentration, and initial water content—defining appropriate parameter ranges is essential. The free swell ratio (FSR) was therefore selected as the primary evaluation criterion. Previous studies have identified fly ash content (*FA*), NaOH concentration (*c*), and initial water content (*w*) as key factors governing the swelling behavior of expansive clays; accordingly, these variables were treated as independently controlled factors in this study. To efficiently quantify their main effects while minimizing experimental effort, a Taguchi orthogonal design was employed. This statistical approach uses orthogonal arrays to systematically evaluate multiple variables and determine their relative contributions to the response through signal-to-noise ratio (SNR) analysis and analysis of variance (ANOVA), and has been widely validated for parameter optimization in geopolymer and materials research [37].

Fly ash, acting as the aluminosilicate precursor, is the primary reactant in the geopolymerization process, and its dosage determines whether the reaction proceeds effectively [1]. Therefore, fly ash content (*FA*) was chosen as the first experimental factor, with three levels: 3%, 6%, and 9%. Due to the inherently low reactivity of fly ash, activation by a strong alkaline solution is necessary. Therefore, NaOH concentration (*c*) was selected as the second factor, with three levels: 2 mol/dm^3^, 4 mol/dm^3^, and 6 mol/dm^3^ [7].

The initial water content (*w*) of the soil significantly influences its mechanical and swelling properties, particularly in high-plasticity clays such as bentonite [11]. Preliminary tests showed that the saturated water content corresponding to a void ratio of *e* = 1.1 was 44%, and specimens with water content below 25% were difficult to shape. Therefore, the initial water content (*w*) was selected, with three levels: 44%, 35%, and 25%. The experimental factors and levels are summarized in Table 1. The corresponding orthogonal experimental design is provided in Table 2, with all samples prepared at a void ratio of *e* = 1.1.

The results from the Taguchi analysis indicated that NaOH concentration exerted the greatest influence on the swelling behavior of the geopolymer-treated soil. To further elucidate this effect, a secondary experimental program was conducted to investigate the relationship between NaOH concentration and swelling characteristics under different initial water contents. The experimental design was summarized in Table 3. All samples were cured for 7 days, with a void ratio of *e* = 1.1 and a constant fly ash content of 9%.

### 2.3. Specimen Preparation and Testing Equipment

Geopolymer-treated soil samples are generally prepared using three main methods [38]: (i) pre-formed binder blending, where an alkali-activated binder paste is prepared first and then blended with separately conditioned wet soil; (ii) the one-part (“just-add-water”) route, where solid NaOH is dry-mixed with the soil–fly ash blend prior to water addition; and (iii) the two-part route, where a NaOH solution is prepared separately and then mixed with the dry soil–fly ash blend. We adopted the two-part route because preliminary trials showed it provides a more homogeneous distribution of the activator/reaction products within the compacted specimens, thereby improving mixture uniformity and test repeatability; it also allows for an accurate control of NaOH concentration.

In this study, the following sample preparation procedure was adopted: Bentonite and fly ash were first oven-dried at 105 °C. The dry materials (fly ash and bentonite) were thoroughly blended, while NaOH pellets were dissolved in distilled water to prepare the alkaline activator. The two mixtures were subsequently combined and mixed using a laboratory mixer, with each mixing stage lasting two minutes and repeated twice to ensure uniformity. The prepared mixture was sealed in a vacuum bag for 24 h to allow complete moisture equilibration. This step homogenizes moisture/alkali distribution and reduces moisture loss and the atmospheric carbonation of NaOH. The bag was evacuated to remove most entrapped air; complete air removal is difficult and was not required for this study.

Samples were then statically compacted to achieve the desired initial void ratio at a controlled loading rate of 0.1 mm/min. Cylindrical specimens with a diameter of 61.8 mm and a height of 20 mm were formed. To facilitate demolding, the inner wall of the mold was coated with a thin layer of petroleum jelly. The compacted samples were sealed in vacuum bags and cured in a controlled environment at 22 ± 2 °C and 70 ± 2% relative humidity until the target curing age was reached. After demolding, the samples were subjected to free swelling tests.

The free swell ratio (FSR) was determined using a free swelling test in accordance with GB/T 50123-2019 [39], which is technically consistent with ISO 17892-13 [40]. The specimen was placed in a rigid confining ring (Nanjing Soil Instrument Factory Co., Ltd., Nanjing, China) to ensure lateral confinement while allowing unrestricted vertical deformation under zero vertical surcharge. Deionized water was used as the soaking fluid to minimize chemical interference from dissolved ions. Upon contact with water, the specimen was allowed to swell freely in the vertical direction.

The test was conducted under controlled laboratory conditions at a temperature of 22 ± 2 °C. The vertical deformation was monitored continuously until the swelling equilibrium was reached. The free swell ratio (FSR) was calculated as

(1) $$\mathrm{FSR}=\frac{H_{t}-H_{0}}{H_{0}}\times 100\%$$

where *H*_0_ is the initial specimen height before soaking, and *H*_t_ is the specimen height at swelling equilibrium.

It should be noted that the solution pH was not actively controlled during soaking. Due to the presence of residual alkali in alkali-activated specimens, the leachate pH may change during immersion. This factor represents a potential limitation and has been acknowledged accordingly.

For the Taguchi orthogonal program, one specimen was prepared and tested for each condition; a three-specimen repeatability verification is provided in Supplementary Materials, Section S7.

X-ray diffraction (XRD) analysis was performed using a powder X-ray diffractometer (Empyrean, Malvern Panalytical, Almelo, The Netherlands). Fourier transform infrared (FTIR) spectroscopy was conducted using an FTIR spectrometer (Nicolet iS50, Thermo Fisher Scientific Inc., Waltham, MA, USA). Scanning electron microscopy (SEM) observations were obtained using a tungsten-filament scanning electron microscope (JSM-IT500, JEOL Ltd., Tokyo, Japan).

## 3. Results and Discussion

### 3.1. Swelling Behavior of Geopolymer-Treated Bentonite

#### 3.1.1. Taguchi Orthogonal Test

Table 4 presents the results of the free swell ratio (FSR) tests. Each value represents the measured response of an individual specimen. The lowest FSR (5.65%) occurred under the conditions of Group 1 (9% fly ash, 6 mol/dm^3^ NaOH, and 44% initial water content), while the highest value (95.85%) was observed in Group 9 (3% fly ash, 2 mol/dm^3^ NaOH, and 35% initial water content), with a nearly 17-fold difference. These results confirm that geopolymer treatment markedly suppresses the swelling of bentonite, and that the effects of individual parameters and their levels are substantial.

Each experimental condition defined by the orthogonal array was tested using one specimen, and the reported free swell ratio (FSR) values correspond to individual measurements rather than averaged values.

To evaluate the relative influence and robustness of each factor, the signal-to-noise ratio (SNR) was calculated using the standard Taguchi formulation:

(2) $$\mathrm{SNR}=-10\log_{10}\left(\frac{1}{n}\sum_{i=1}^{n}y_{i}^{2}\right)$$

where *y_i_* represents the measured free swell ratio. As swelling suppression was the objective, the smaller-the-better criterion was adopted. The SNR provides a quantitative measure of factor influence and sensitivity.

**Figure 1:**
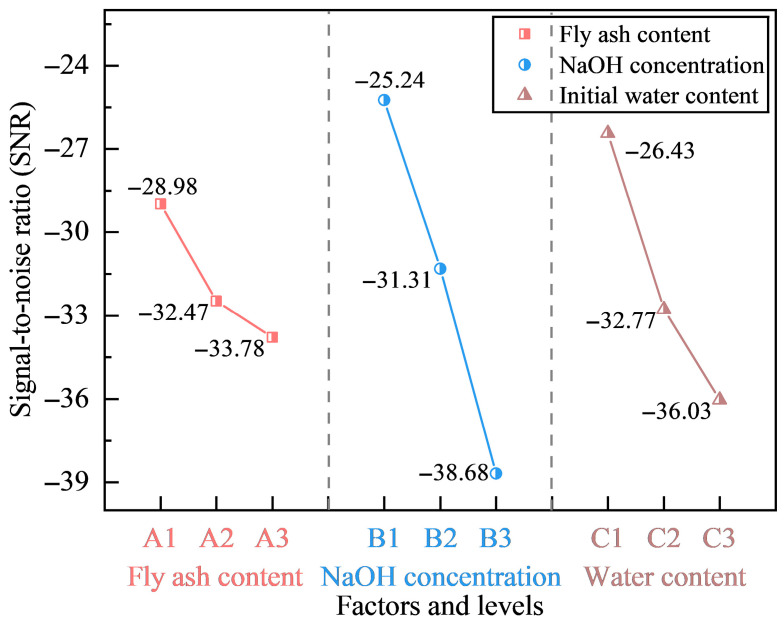
The main effect of signal-to-noise ratio (SNR) for different factors.

The influence of each parameter on swelling behavior was further evaluated using the signal-to-noise ratio (SNR). The main effects plot of SNR is shown in Figure 1. A greater variation in SNR indicates a stronger factor influence. The results show that NaOH concentration exerts the strongest control on swelling behavior, followed by the initial water content and fly ash content. This confirms that alkaline activation is the dominant factor governing swelling suppression.

**Figure 2:**
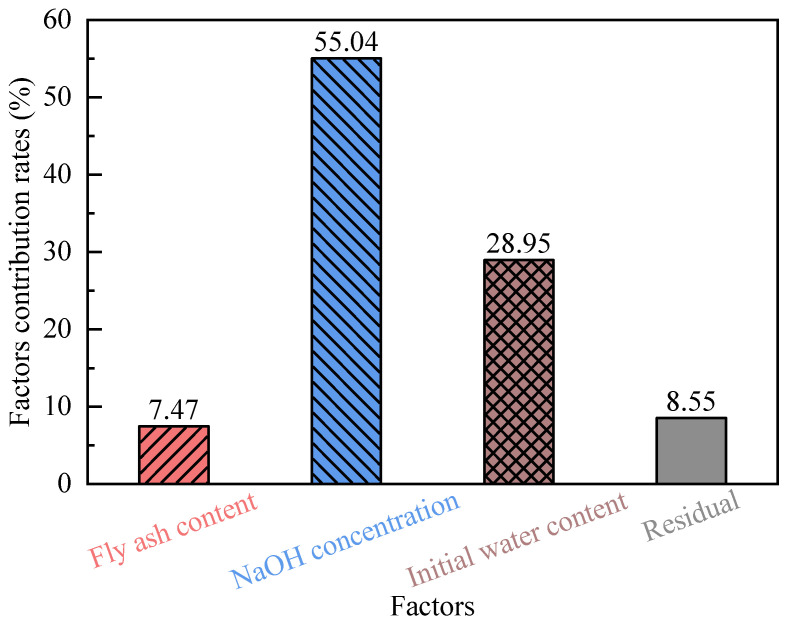
Contribution rates of signal to noise ratio for different factors.

Figure 2 quantifies the percentage contributions of key factors influencing the free swell ratio (FSR) of modified bentonite, calculated using the standardized contribution rate formula:

(3) $${\mathrm{Contribution}\;\mathrm{Rate}}_{i}=\left(\frac{{\mathrm{SS}}_{i}}{{\mathrm{SS}}_{\mathrm{Total}}}\right)\times 100\%$$

where SS*_i_* represents the Sum of Squares for the *i*-th factor; SS_Total_ represents the Total Sum of Squares.

As shown in Figure 2, the relative contributions of each factor to the reduction in the free swell ratio (FSR) follow the order: NaOH concentration (55.04%) > initial water content (28.95%) > fly ash content (7.47%). This quantitative hierarchy is consistent with the conclusions presented in Section S3 of Supplementary Materials, reinforcing that NaOH concentration is the primary parameter governing the swelling suppression of geopolymer-treated bentonite.

#### 3.1.2. NaOH Concentration Effect on Free Swell Ratio (FSR) of the Modified Bentonite Clay

**Figure 3:**
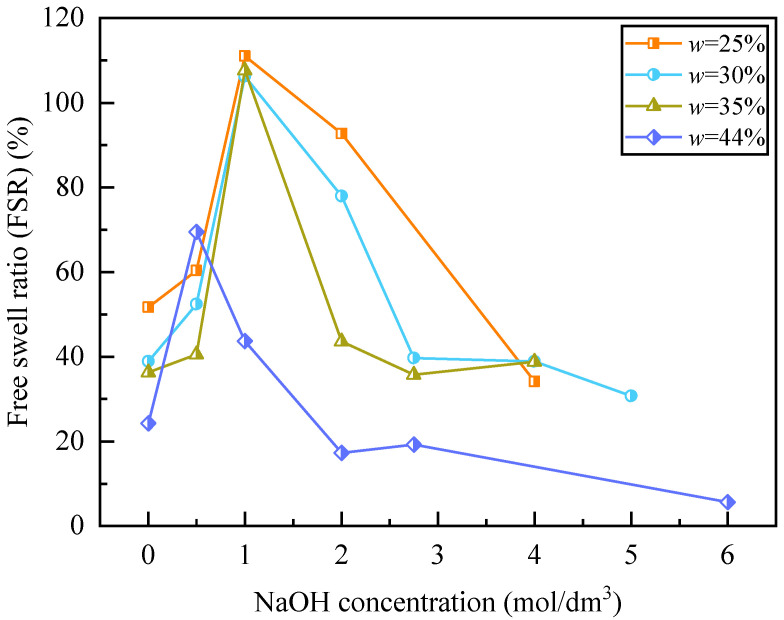
Dependence of free swell ratio (FSR) on NaOH concentration in geopolymer-treated bentonite at varying initial water contents.

The swelling behavior of geopolymer-modified soils exhibits a strong dependence on NaOH concentration, following a distinct two-stage “enhancement–inhibition” pattern (Figure 3). In the low-concentration range (0–1 mol/dm^3^), the free swell ratio (FSR) increases rapidly with alkali concentration, reaching peak values of 104.8–111.0% for unsaturated specimens (*w* = 25–35%), as determined from the measured specimen height change during the standardized free swelling test described in Section 2.3 and calculated using Equation (1). These values are approximately 2.5–4 times higher than those of untreated soil (*c* = 0). Beyond a critical concentration of 3 mol/dm^3^, the FSR decreases sharply to 15–40% below the untreated level and subsequently stabilizes with minimal variation. A comparable conclusion was likewise reached in Section S4 of Supplementary Materials.

The interaction between alkali activation and bentonite minerals evolves progressively with an increasing NaOH concentration, shifting from interlayer cation exchange-dominated swelling to dissolution-assisted gel-controlled stabilization. At low concentrations (≤1 mol/dm^3^), Na^+^–Ca^2+^ interlayer exchange dominates swelling; although gel formation may initiate, XRD shows only a faint amorphous shoulder/hump, indicating insufficient gel to restrain interlayer expansion (as demonstrated in Section 3.2.1). At intermediate concentrations (1–3 mol/dm^3^), OH^−^ ions partially dissolve the montmorillonite framework, releasing reactive Si and Al species that both weaken the original layered structure and initiate geopolymer gel nucleation within the pore network.

At higher concentrations (>3 mol/dm^3^), extensive aluminosilicate dissolution supplies sufficient precursors for alkali-activated polymerization, resulting in substantial geopolymer gel precipitation within pore spaces and along clay particle surfaces. The gel fills voids, binds clay particles, and produces a denser matrix, while the elevated ionic strength compresses the diffuse double layer. Consequently, the dominant mechanism shifts from diffuse-double-layer-controlled swelling to gel-induced structural densification and mineral–gel composite stabilization.

The moisture content significantly influences swelling behavior by affecting ion transport, water uptake, and geopolymerization kinetics. Under a high initial water content, continuous pore water facilitates ion mobility; however, the hydration and expansion of montmorillonite layers increase the diffusion path tortuosity and reduce the additional water absorption capacity. As the alkali concentration increases, enhanced dissolution and geopolymer gel formation stabilize the soil structure and suppress swelling. This stabilized gel–clay composite structure limits interlayer expansion and reduces moisture sensitivity, leading to a stabilized free swell ratio (FSR) of approximately 60–80% of the untreated condition. Therefore, swelling behavior is governed by the coupled effects of ion transport, mineral dissolution, and geopolymer gel formation rather than ion mobility alone.

### 3.2. Modification Mechanism of Geopolymer for Bentonite Clay

#### 3.2.1. X-Ray Diffraction (XRD) Analysis

**Figure 4:**
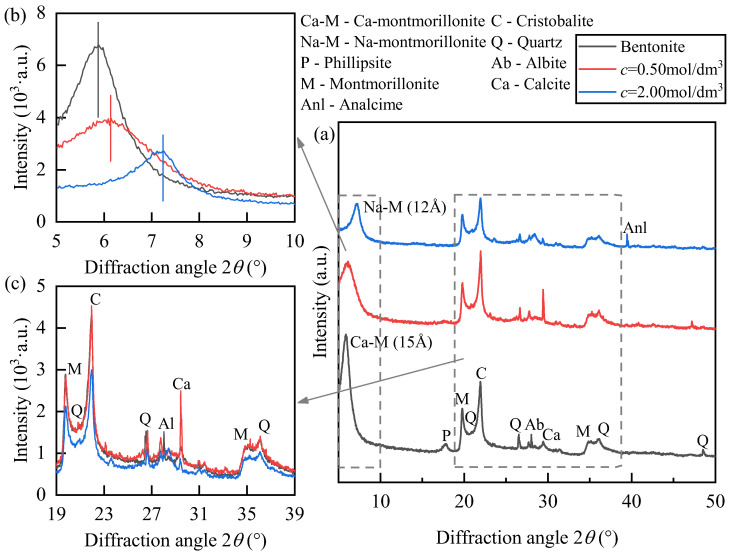
XRD patterns of NaOH-treated soil: Concentration effects at *w* = 25% initial water content. (**a**) Overall diffraction pattern (5–50°); (**b**) Enlarged view of the low-angle region (5–10°); (**c**) Enlarged view of the main diffraction region (19–39°).

X-ray diffraction (XRD) analyses were performed on geopolymer-treated soil samples with an initial water content of *w* = 25% and NaOH concentrations of 0.50 mol/dm^3^ and 2.00 mol/dm^3^. A comparative test was also performed on untreated bentonite with the same water content, as shown in Figure 4. In Figure 4a, the characteristic phillipsite (P) peak in the untreated bentonite is absent in both alkali-treated samples, indicating that the strong alkali–silica–alumina reaction dissolved the phillipsite and re-precipitated it as other crystalline or amorphous geopolymeric phases.

In Figure 4b, the Ca-montmorillonite (Ca-M) peak (d ≈ 15 Å, 2θ ≈ 6°) is most pronounced in the untreated sample. At 0.50 mol/dm^3^, the peak intensity decreases significantly, indicating a partial replacement of interlayer Ca^2+^ by Na^+^. At 2.00 mol/dm^3^, the Ca-M peak is replaced by the Na-M peak, and the interlayer spacing decreases from 14.379 Å to 12.266 Å, with a further contraction to 12.044 Å at higher concentration, accompanied by a reduced peak intensity. These quantitative changes provide evidence of Ca^2+^–Na^+^ interlayer exchange and progressive structural reorganization associated with alkali activation. Such changes alter interlayer bonding and hydration behavior, which contributes to the observed increase in swelling at intermediate alkali concentrations and subsequent reduction at higher concentrations due to structural destabilization and geopolymer gel formation. This interpretation is also consistent with the FTIR results showing a weakening of Si–O–Si and Al–OH related bands, indicating mineral dissolution and structural reorganization.

In Figure 4c, the diffraction peaks of quartz (Q), cristobalite (C), and albite (Ab) remain similar to those of untreated soil at 0.50 mol/dm^3^. However, at 2.00 mol/dm^3^, these peaks weaken and broaden, indicating that OH^−^ ions dissolve high SiO_2_ phases and promote geopolymerization. A broad hump in the 2θ = 27–29° range reflects the formation of amorphous N-A-S-H (sodium–aluminosilicate-hydrate) gel. A faint shoulder at 0.50 mol/dm^3^ and a distinct hump at 2.00 mol/dm^3^ indicate progressive gel formation with increasing alkalinity. Additionally, a new diffraction peak near 2θ ≈ 40° corresponds to analcime (Anl), confirming the recrystallization of dissolved Si and Al species under high-alkali conditions.

Overall, at 0.50 mol/dm^3^ NaOH, Ca^2+^ replacement and gel formation are limited, and the mineral framework remains largely intact, leading to minimal swelling behavior change. At 2.00 mol/dm^3^, the Ca-M to Na-M transformation is nearly complete, SiO_2_ phases are activated, and amorphous gel and analcime formation enhance structural bonding. However, the stronger swelling capacity of Na-M produces the complex behavior of swelling initially increasing and then decreasing.

**Figure 5:**
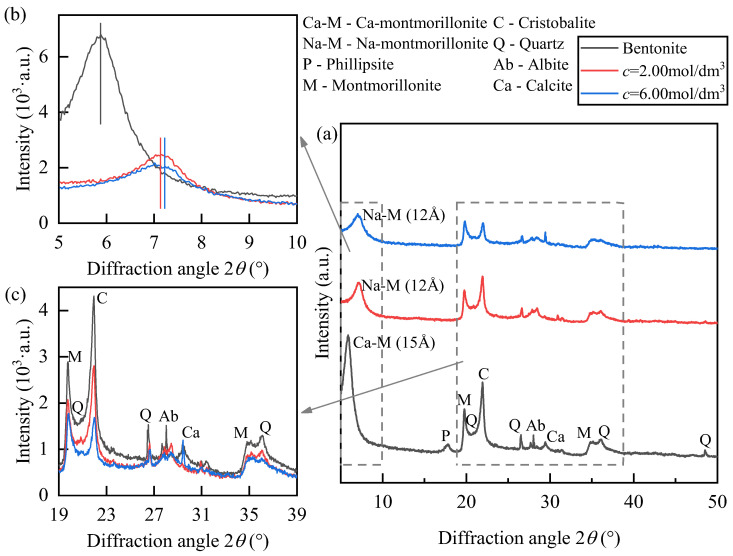
XRD patterns of NaOH-treated soil: concentration effects at *w* = 44% initial water content. (**a**) Overall diffraction pattern (5–50°); (**b**) Enlarged view of the low-angle region (5–10°); (**c**) Enlarged view of the main diffraction region (19–39°).

XRD tests were also conducted on specimens with *w* = 44% and NaOH concentrations of 2.00 mol/dm^3^ and 6.00 mol/dm^3^ (Figure 5). As shown in Figure 5a, the phillipsite (P) peak disappears in both treated soils, confirming its dissolution and re-precipitation as observed previously.

As shown in Figure 5b, the Ca-M peak (d ≈ 15 Å, 2θ ≈ 6°) of untreated bentonite shifts to 2θ ≈ 7.1° (d ≈ 12.351 Å) after treatment with 2.00 mol/dm^3^ NaOH, accompanied by a reduced intensity. At 6.00 mol/dm^3^, the d-spacing further contracts to 12.044 Å with additional peak weakening. These quantitative changes in basal spacing and peak intensity confirm the progressive interlayer cation exchange and structural modification. The resulting changes in interlayer electrostatic interactions and hydration capacity directly influence macroscopic swelling behavior. At higher alkali concentrations, geopolymer gel formation and increased ionic strength further stabilize the structure and suppress swelling [41].

Figure 5c shows the progressive attenuation of quartz, cristobalite, and albite peaks with increasing alkalinity, indicating the dissolution of SiO_2_-rich phases and re-precipitation as gels. However, the diffuse hump near 2θ = 27–29° is less intense at 6.00 mol/dm^3^ than at 2.00 mol/dm^3^, suggesting that excessive alkalinity may degrade gel phases and weaken the crystalline framework, thereby decreasing swelling. In summary, high NaOH concentrations (*c* = 6 mol/dm^3^) compress the double layer through strong ionic effects, reducing interlayer spacing and causing crystal collapse, which lowers the macroscopic swelling rate.

Under a high water content (*w* = 44%), Na^+^ migration and solute diffusion are enhanced, accelerating ion exchange and gelation but also promoting structural collapse and gel degradation in highly alkaline environments. Consequently, the swelling behavior follows a pattern of “initial increase followed by reduction.” The interplay between NaOH concentration and water content governs the balance among interlayer ion exchange, crystal stability, and amorphous gel formation, which together determine the microstructural evolution and macroscopic swelling response of NaOH-activated geopolymer soils.

**Figure 6:**
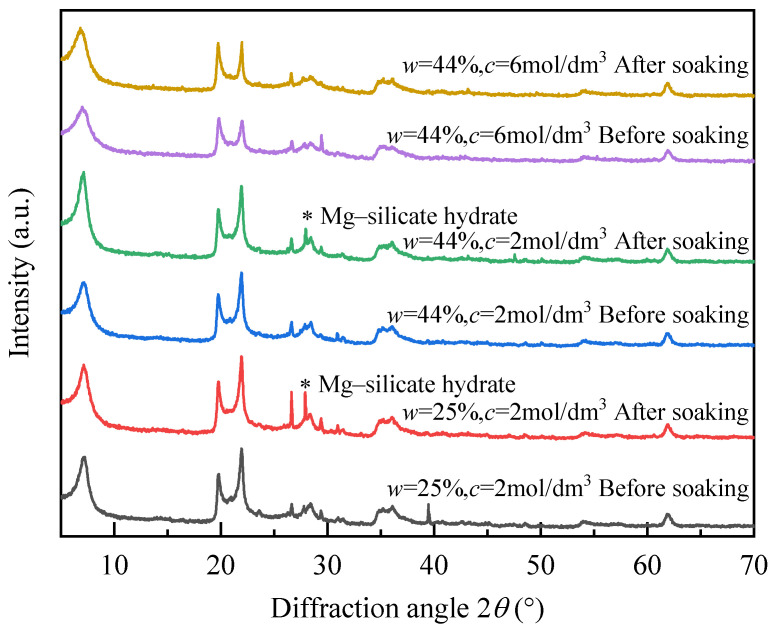
XRD patterns of the samples before and after soaking. The asterisk (*) indicates the diffraction peaks attributed to magnesium silicate hydrate phases.

To further elucidate the swelling behavior, XRD analyses were performed before and after soaking (Figure 6). For the samples with *w* = 25%, *c* = 2 mol/dm^3^ and *w* = 44%, *c* = 2 mol/dm^3^, additional diffraction peaks appeared after soaking (marked by *), which are consistent with the formation of magnesium silicate hydrate phases under alkaline conditions. This likely results from the release of Mg^2+^ from montmorillonite interlayers and its reaction with dissolved silicate species during alkali activation and subsequent soaking. The peak positions are comparable to those reported for sepiolite; however, this assignment remains tentative, and the observed features may also correspond to other magnesium silicate hydrate phases. Therefore, these results indicate the probable formation of magnesium silicate phases, possibly including sepiolite, associated with alkali-induced mineral transformation. Such mineralogical modifications provide direct evidence of structural reorganization and help explain the reduction in swelling at higher alkali concentrations, as observed in Figure 3.

#### 3.2.2. Fourier Transform Infrared Spectroscopy (FTIR) Analysis

**Figure 7:**
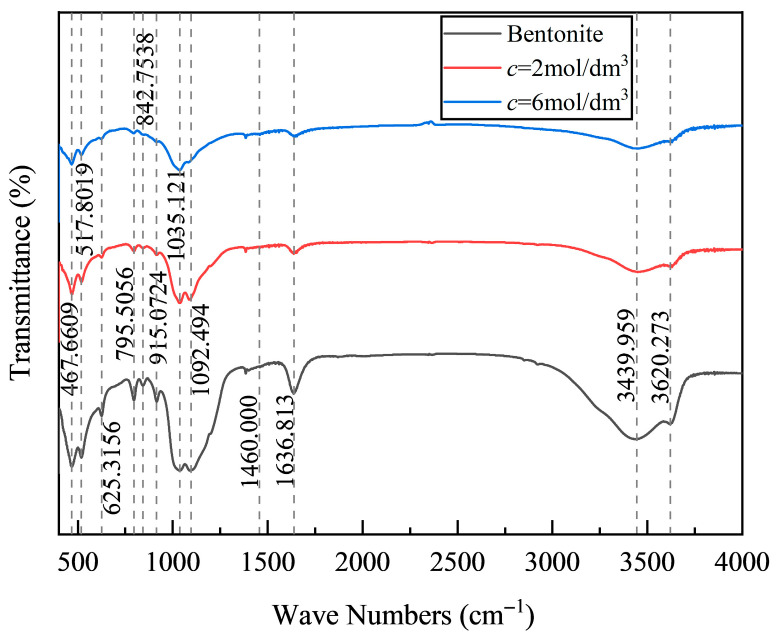
FTIR spectra of samples with an initial water content of 44% at different concentrations.

As shown in Figure 7, the FTIR spectra demonstrate that increasing the NaOH concentration from 0 to 6 mol/dm^3^ induces a progressive transformation in montmorillonite, characterized by ion exchange, dissolution, reprecipitation, and framework reconstruction. The absorption bands at 467 cm^−1^ (Si–O–Si bending vibration), 517 cm^−1^ (Si–O–Mg bending vibration), and 915 cm^−1^ (Al–O–H bending vibration) weaken markedly and nearly disappear with an increasing NaOH concentration. This attenuation indicates that Na^+^ ions replace interlayer cations, facilitating the leaching of Mg^2+^, Al^3+^, and Si^4+^. The released Mg^2+^ may react with dissolved silicate species to form Mg–silicate hydrate phases (possibly sepiolite-like).

At a high alkalinity (*c* = 6 mol/dm^3^), the disappearance of the 1092 cm^−1^ band (Si–O–Si stretching vibration) suggests the depolymerization and repolymerization of silicate tetrahedra. Part of the Si–O–Si framework is disrupted and reconstituted into N–A–S–H or other aluminosilicate gels, signifying a substantial chemical transformation of the matrix. This structural reorganization converts the layered silicate framework into a three-dimensional geopolymeric network, effectively reducing the soil’s swelling potential [42].

The absorption peaks at 1636 cm^−1^ (H–O–H bending vibration) and 3439 cm^−1^ (interlayer water stretching vibration) also decrease significantly as the NaOH concentration increases. This indicates that interlayer water is either encapsulated within newly formed gels or expelled from the system, while hydroxyl groups actively participate in polymerization [34,43]. Consequently, the material becomes progressively denser, with a reduced water absorption capacity and modified interlayer characteristics that suppress subsequent swelling. Similarly, the 3620 cm^−1^ peak (Al–OH stretching vibration) weakens under elevated NaOH concentrations, signifying the disruption of octahedral Al–OH bonds and dissolution of Al^3+^ into the pore solution, where it contributes to geopolymer network formation.

So, NaOH activation profoundly modifies the chemistry and structure of montmorillonite. The systematic weakening and disappearance of characteristic absorption peaks confirm the processes of ion exchange, mineral dissolution, and secondary phase formation (e.g., sepiolite). These transformations intensify with increasing NaOH concentration, illustrating that strong alkali activation effectively converts the montmorillonite–fly ash system into a stable, low-swelling, and high-strength geopolymer composite.

#### 3.2.3. Scanning Electron Microscopy (SEM) Analysis

**Figure 8:**
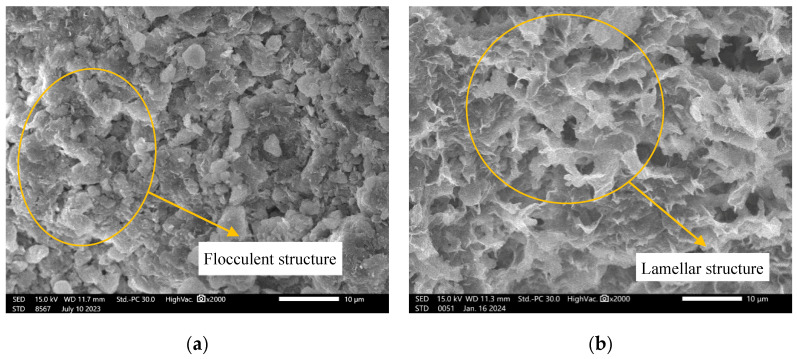
SEM micrographs of untreated bentonite before and after soaking. (**a**) Before soaking; (**b**) after soaking.

Scanning electron microscopy (SEM) analyses were conducted on untreated samples before and after water soaking. Figure 8a presents the compacted sample prior to soaking (*w* = 25%), whereas Figure 8b illustrates the microstructure after soaking (*w* = 82.66%). Before soaking, when the crystal layers contain little or no interlayer water, the layers are held together primarily by weak van der Waals forces. The structure appears dense and flocculent, with a uniform, closely packed arrangement. After soaking, the sample absorbs substantial water, leading to a significant increase in interlayer spacing and the formation of a clearly defined layered crystal structure. The extremely thin crystal plates exhibit pronounced expansion, explaining the marked increase in the macroscopic free swell ratio (FSR) after saturation.

**Figure 9:**
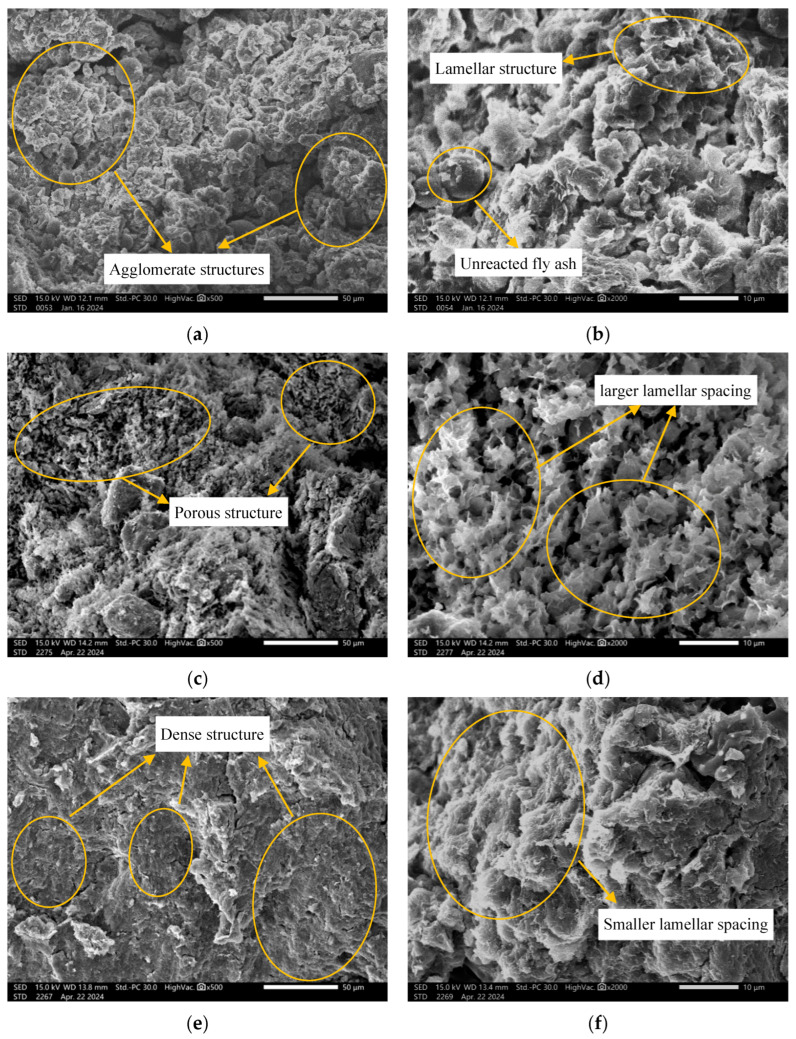
SEM images of geopolymer-treated soil samples after soaking. (**a**) *c* = 0.50 mol/dm^3^ (×500); (**b**) *c* = 0.50 mol/dm^3^ (×2000); (**c**) *c* = 2.00 mol/dm^3^ (×500); (**d**) *c* = 2.00 mol/dm^3^ (×2000); (**e**) *c* = 6.00 mol/dm^3^ (×500); (**f**) *c* = 6.00 mol/dm^3^ (×2000).

Figure 9 shows the SEM images of geopolymer-treated samples after soaking, corresponding to NaOH concentrations of *c* = 0.50 mol/dm^3^, 2.00 mol/dm^3^, and 6.00 mol/dm^3^. The initial water contents prior to soaking were *w* = 25%, 25%, and 44%, while the post-soaking water contents reached *w* = 72.44%, 102.88%, and 58.42%, respectively.

At *c* = 0.50 mol/dm^3^ (Figure 9a,b), the microstructure exhibits distinct agglomerates resulting from geopolymer gel formation, which binds the montmorillonite particles. However, due to the low OH^−^ concentration, some fly ash remains unreacted, and numerous spherical glassy particles are still visible. At a higher magnification (2000×), the composite microstructure of montmorillonite, residual fly ash, and gel products can be clearly observed, forming a partially bonded, interwoven network.

When the NaOH concentration increases to *c* = 2.00 mol/dm^3^, the microstructure undergoes notable transformation (Figure 9c,d). At 500× magnification, two major differences are evident: (1) unreacted fly ash particles are nearly absent, indicating a more complete geopolymerization; and (2) the structure becomes more porous. At this concentration, calcium montmorillonite is largely converted to sodium montmorillonite, which exhibits greater hydration and expanded interlayer spacing, thereby enhancing permeability and providing channels for water migration [44].

At a higher concentration (*c* = 6.00 mol/dm^3^), as shown in Figure 9e,f, the microstructure becomes denser after soaking. Excess alkali induces dissolution and reprecipitation within the agglomerates, followed by interlayer compression and reduced swelling potential. The montmorillonite appears as coiled, thin sheets with fewer voids and a tighter arrangement, indicating decreased interlayer spacing and a reduction in overall expansivity.

#### 3.2.4. Mechanism Analysis of Geopolymer Treatment of Expansives

**Figure 10:**
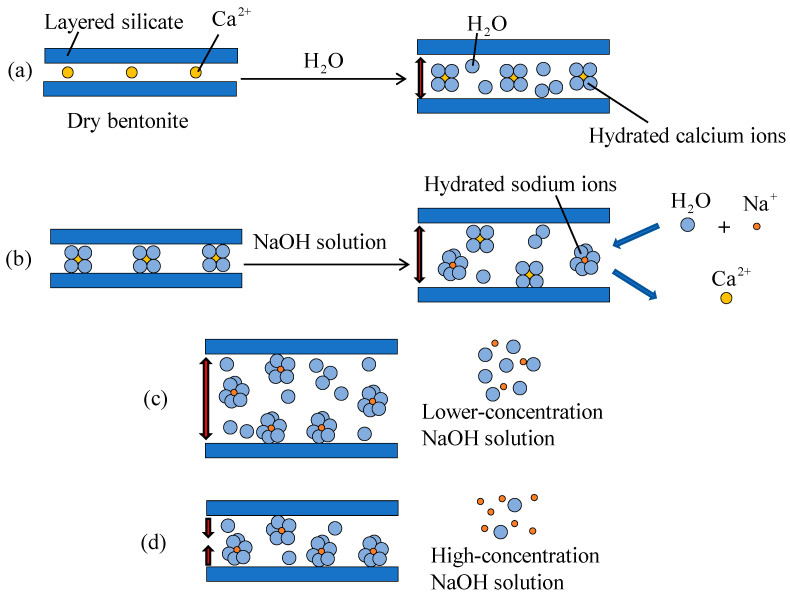
Schematic of the mechanism of OH- action in geopolymer treatment of soil. (**a**) Hydration and interlayer expansion of Ca-bentonite; (**b**) Possible Ca^2+^–Na^+^ cation exchange in NaOH solution; (**c**) Increased interlayer spacing at low NaOH concentration; (**d**) Reduced interlayer spacing at high NaOH concentration.

When montmorillonite interacts with water, its hydration-induced expansion occurs in two sequential stages before the onset of secondary expansion [45]. The first stage, surface hydration, involves the attraction of water molecules into the interlayer spaces by the residual valence forces of functional groups and interlayer cations (Ca^2+^). This results in the formation of hydrated calcium ions, which increases the interlayer spacing. The second stage, osmotic expansion, arises because the concentration of interlayer cations (Ca^2+^) is much higher than that in the external solution. The resulting osmotic pressure gradient draws additional water molecules into the interlayer, further enlarging the spacing between layers (Figure 10a). This osmotic process is the dominant mechanism of montmorillonite swelling and contributes to an expansion over 20 times greater than that caused by surface hydration [45].

When montmorillonite is treated under alkaline activation, interlayer Ca^2+^ is progressively replaced by Na^+^ from the NaOH solution, forming sodium montmorillonite (Figure 10b), while concurrent aluminosilicate dissolution supplies reactive species for subsequent gel formation, as evidenced by the XRD results discussed earlier. With an increasing NaOH concentration, Na^+^ ions continue to exchange with Ca^2+^, leading to a reduction in calcium montmorillonite and a corresponding increase in sodium montmorillonite. Consequently, the macroscopic free swell ratio (FSR) of the sample rises steadily. Once the NaOH concentration reaches a critical threshold, most Ca^2+^ ions are replaced by Na^+^, and ion exchange reaches saturation, yielding the maximum FSR (Figure 10c). At higher NaOH concentrations, excess Na^+^ ions elevate ionic strength and compress the diffuse double layer, while simultaneous geopolymer gel precipitation fills interparticle voids and binds clay platelets, collectively diminishing interlayer expansion and water uptake (Figure 10d). This behavior explains the observed transition from swelling enhancement to inhibition at elevated alkali concentrations.

## 4. Conclusions

This study quantitatively evaluated the swelling response of alkali-activated fly ash–bentonite within the tested ranges of fly ash content (*FA* = 3–9%), NaOH concentration (*c* = 0–6 mol/dm^3^), and initial water content (*w* = 25–44%), with specimens compacted to an initial void ratio of *e* = 1.1 under controlled curing conditions. The key quantitative findings and engineering implications are summarized as follows:

(1) Taguchi analysis demonstrated that NaOH concentration is the dominant parameter controlling swelling behavior, contributing 55.04% of the total variation, followed by the initial water content (28.95%) and fly ash content (7.47%). This establishes alkaline activation level as the primary design variable governing expansive deformation.

(2) Across the extended concentration range (*c* = 0–6 mol/dm^3^), the free swell ratio (FSR) exhibited a distinct two-stage trend. In the low-alkali regime (*c* ≤ 1 mol/dm^3^), Na^+^-induced interlayer exchange increased FSR to peak values of 104.8–111.0%, approximately 2.5–4.0 times higher than the untreated condition. A critical threshold was identified at *c* ≈ 3 mol/dm^3^, beyond which swelling was effectively suppressed and stabilized. Under the orthogonal design conditions, the minimum FSR reached 5.65% at *FA* = 9%, *c* = 6 mol/dm^3^, and *w* = 44%, confirming the strong effectiveness of alkali activation in mitigating expansive deformation.

(3) From an engineering perspective, deformation control is the primary performance requirement for expansive soil subgrades, as swelling-induced heave and cracking typically govern serviceability, whereas untreated bentonite often already satisfies basic bearing capacity requirements. Therefore, swelling suppression represents the critical criterion for ensuring structural stability. As a complementary mechanical indicator, the unconfined compressive strength (UCS) results (Supplementary Materials, Section S6) show that alkali activation also improves strength, with peak UCS values of approximately 1.41 MPa (*w* = 35%) and 1.20 MPa (*w* = 44%) at *c* ≈ 2 mol/dm^3^, confirming that swelling reduction is accompanied by enhanced mechanical integrity.

Overall, the identified critical NaOH threshold (*c* ≈ 3 mol/dm^3^) provides a practical design reference for expansive soil stabilization, enabling effective swelling suppression while maintaining or improving mechanical stability. These findings support the use of alkali-activated fly ash treatment as an effective strategy for deformation control and the long-term serviceability of expansive soil subgrades.

## Figures and Tables

**Table 1:** Factors and levels considered in Taguchi orthogonal design experiment.

Factors	ID	Levels		
		1	2	3
Fly ash content (*FA*)	A	9%	6%	3%
NaOH concentration (*c*)	B	6 mol/dm^3^	4 mol/dm^3^	2 mol/dm^3^
Initial water content (*w*)	C	44%	35%	25%

**Table 2:** Detailed Taguchi orthogonal design experiments.

No.	A	B	C	Fly Ash Content (%)	NaOH Concentration (mol/dm3)	Initial Water Content (%)	Temperature (℃)	Relative Humidity (%)
1	1	1	1	9	6	44	22 ± 2	70 ± 2
2	1	2	2	9	4	35	22 ± 2	70 ± 2
3	1	3	3	9	2	25	22 ± 2	70 ± 2
4	2	1	2	6	6	35	22 ± 2	70 ± 2
5	2	2	3	6	4	25	22 ± 2	70 ± 2
6	2	3	1	6	2	44	22 ± 2	70 ± 2
7	3	1	3	3	6	25	22 ± 2	70 ± 2
8	3	2	1	3	4	44	22 ± 2	70 ± 2
9	3	3	2	3	2	35	22 ± 2	70 ± 2

**Table 3:** Testing program considering the effect of NaOH concentration on the performance of geopolymer-treated expansive soil.

Initial Water Content (%)	NaOH Concentration (mol/dm^3^)
25, 30, 35, 44	0, 0.50, 1.00, 2.00, 2.75, 4.00, 6.00

**Table 4:** Results of the free swell ratio (FSR) tests.

No.	Fly Ash Content (%)	NaOH Concentration (mol/dm^3^)	Initial Water Content (%)	Free Swell Ratio (FSR) (%)
1	9	6	44	5.65
2	9	4	35	38.85
3	9	2	25	92.70
4	6	6	35	20.27
5	6	4	25	51.32
6	6	2	44	71.35
7	3	6	25	53.28
8	3	4	44	22.83
9	3	2	35	95.85

## Data Availability

The original contributions presented in the study are included in the article/Supplementary Materials, further inquiries can be directed to the corresponding authors.

## Supplementary Materials

The following supporting information can be downloaded at: https://www.mdpi.com/article/10.3390/polym18050606/s1, Figure S1: Particle size distribution of the bentonite; Figure S2: XRD results of the fly ash; Figure S3: Relationship between free swell ratio (FSR) response index and the levels of different factors; Figure S4: Free swell ratio (FSR)-time relationships in semi-log scale: NaOH concentration dependence at varied initial water contents under zero-loading; Figure S5: Primary swelling coefficient as a function of NaOH concentration at varying initial water contents; Figure S6: Crystallized sodium carbonate on the surface of permeable stone and corresponding XRD pattern; Figure S7: Evolution of unconfined compression strength (UCS) with NaOH concentration for geopolymer treated bentonite under different initial water contents; Figure S8. Time-dependent swelling curves of parallel Free Swell Ratio (FSR) specimens. References [46,47] are cited in the supplementary materials.
