# Supplementary material for: Effect of Alkali Activation on Swelling Suppression and Microstructural Development in Geopolymer-Stabilized Bentonite
Source: Polymers (Basel). 2026 Feb 28;18(5):606. doi: 10.3390/polym18050606 (PMC12987071; doi:10.3390/polym18050606)
Supplement: Supplementary file 1 [file polymers-18-00606-s001.zip › polymers-4160927-supplementary.pdf]

Supplementary Material for "Effect of Alkali Activation on Swelling Suppression and Microstructural Development in Geopolymer-Stabilized Bentonite"

Tengshen Jing[1,2], Shengyang Yuan[1,2,*], Xianfeng Liu[1,2,*], Yulin Liu[1,2], Haibin Xu[1,2], Weixing Zhou[1,2], Pengjie Lin[1,2,3], Guanlu Jiang[1,2]

[1] Key Laboratory of High-speed Railway Engineering of Ministry of Education, Southwest Jiaotong University, Chengdu 610031, China

[2] School of Civil Engineering, Southwest Jiaotong University, Chengdu 610031, China

[3] School of Civil Engineering, Xinjiang Institute of Engineering, Urumqi 830023, China

* Correspondence: shengyang.yuan@swjtu.edu.cn (S. Yuan); Xianfeng.liu@swjtu.edu.cn (X. Liu)

Contents of This File: The supplementary materials provide additional experimental evidence, including the particle size distribution of the commercial bentonite, the XRD characterization of the fly ash, the sensitivity analysis of key parameters, the effect of NaOH concentration on the free swell ratio (FSR), and the formation of sodium hydroxide deposits on the porous stone.

Number of Sections: 7 (S1-S7)

Number of Figures: 8 (Figure S1- Figure S8)

Number of Equations: 1 (S.1)

S1. Particle Size Characteristics of the Commercial Bentonite Used in This Study

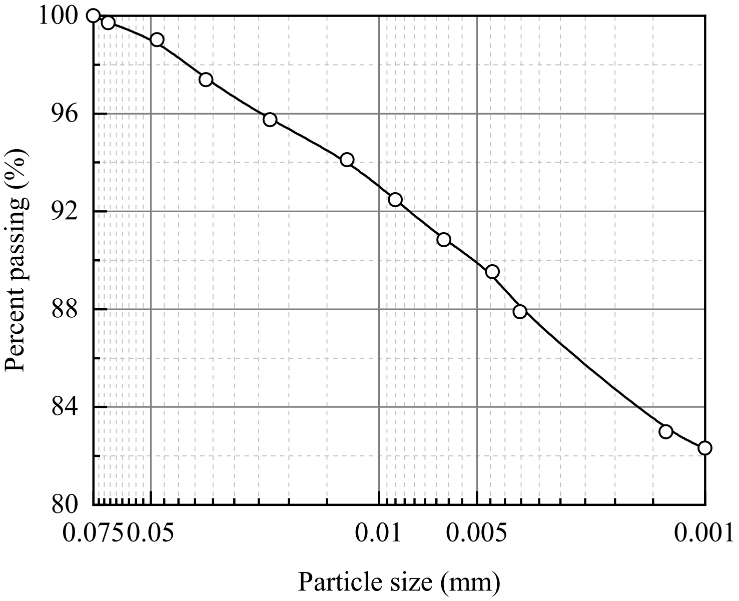

Figure S1. Particle size distribution of the bentonite.

The particle size distribution of the commercial montmorillonite used in this study was determined using the hydrometer method. Based on the particle size distribution curve shown in Figure S1, the commercial bentonite sample exhibits a predominantly fine-grained composition. The entire sample passes the 0.075 mm sieve, indicating the absence of sand-sized particles. The gradation curve shows a smooth and continuous decrease in percent passing with decreasing particle size, suggesting a well-graded distribution within the silt and clay fractions.

In the silt-size range (0.075–0.005 mm), the percent passing decreases modestly from approximately 100% to about 90%. A more pronounced reduction is observed in the clay-size range (<0.005 mm), where the percent passing decreases further to approximately 82% at a particle size of 0.001 mm. This indicates that the bentonite contains a substantial proportion of clay-sized particles, which is consistent with the typical characteristics of montmorillonite-rich bentonite. The high clay content also explains the material's high liquid limit and strong swelling potential.

S2. X-ray Diffraction (XRD) Analyses of the Commercial Fly Ash Used in This Study

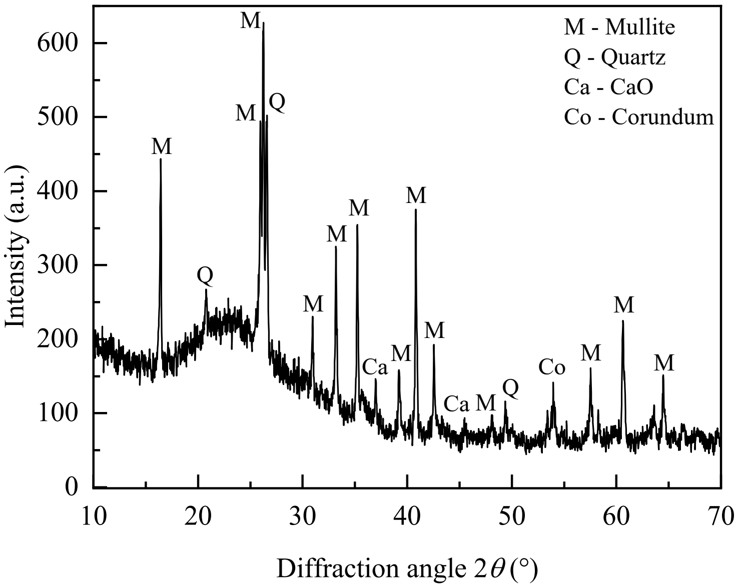

Figure S2. XRD results of the fly ash.

The XRD pattern of the fly ash (Figure S2) shows that mullite is the predominant crystalline phase, as indicated by several strong reflections between 16° and 36° and additional peaks up to 60°. Quartz is also clearly present, with characteristic peaks near 20.8°, 26.6°, and 50°. Minor diffraction peaks assigned to CaO and corundum appear around 37°–38°, 47°, and 52°, respectively. In addition, the broad hump between 15° and 35° suggests the presence of an amorphous aluminosilicate glass phase.

Overall, the XRD results indicate that the fly ash consists mainly of mullite and quartz, accompanied by small amounts of CaO and corundum, as well as a significant amorphous phase. This combination of crystalline and amorphous components is typical of coal-derived fly ash and provides a suitable mineralogical basis for geopolymerization.

S3. Sensitivity Analysis of Key Parameters

To quantify the influence of each parameter, a response index was defined as the mean free swell ratio (FSR) corresponding to each factor level. For example, for fly ash

content, level 1 (9%) represents the average FSR of Groups 1–3. The calculated response indices for all factors are presented in Figure S3. Increasing the fly ash content from level 3 (3%) to level 1 (9%) produced an approximately linear reduction of about 25% (≈ 5.8 percentage points per level), demonstrating a steady inhibitory effect. This decreasing trend reflects the progressive pozzolanic interaction between fly ash and the expansive clay matrix, indicating that fly ash acts as a secondary yet consistently effective stabilizing factor.

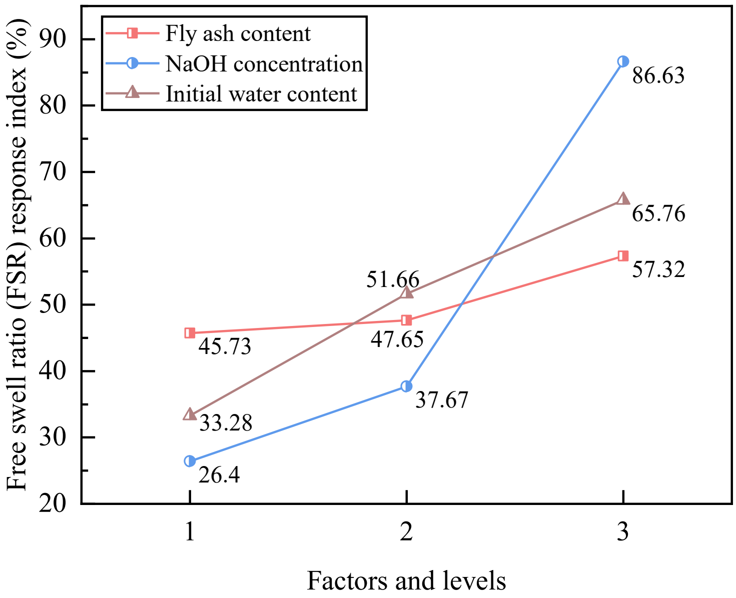

Figure S3. Relationship between free swell ratio (FSR) response index and the levels of different factors.

In contrast, NaOH concentration exhibited the strongest sensitivity among all variables. Lowering the concentration from 6 mol/dm^3 (level 3) to 2 mol/dm^3 (level 1) led to a dramatic 228% increase in the response index, corresponding to a sensitivity coefficient of -60.23% per level. This reinforces that alkaline strength is the dominant control parameter for swelling suppression, primarily through its role in promoting geopolymerization and densifying the soil–binder microstructure. Initial water content also exerted a marked influence: reducing the water content from 44% to 25% increased the response index by roughly 98%, emphasizing the coupled significance of moisture

regulation and chemical activation in governing the swelling behavior of treated bentonite.

S4. NaOH Concentration Effect on Free Swell Ratio (FSR) of the Modified Bentonite Clay

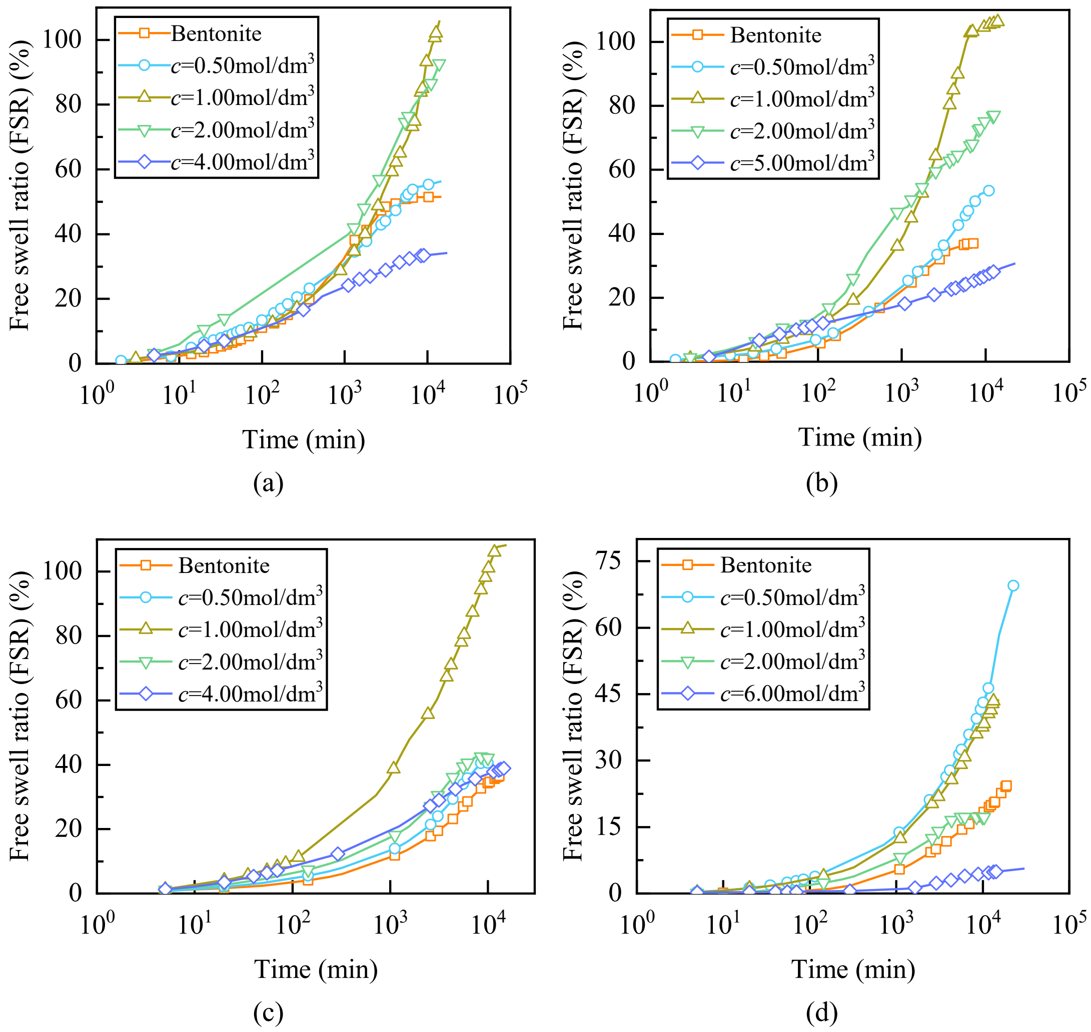

Figure S4. Free swell ratio (FSR)-time relationships in semi-log scale: NaOH concentration dependence at varied initial water contents under zero-loading. (a) w = 25%; (b) w = 30%; (c) w = 35% (d) w = 44%.

Semi-logarithmic swelling–time curves (Figure S4) confirm that bentonite swelling progresses through three characteristic stages under all water contents. In the initial stage, specimens with different NaOH concentrations exhibit nearly identical swelling rates due to comparable initial dry density and pore volume. During the

primary swelling stage, higher final free swell ratio (FSR) correspond to higher swelling rates. As NaOH concentration increases to 1 mol/dm^3, enhanced Na^+ exchange intensifies swelling, but further concentration increases reduce the rate. At high concentrations (⩾ 5 mol/dm^3), the swelling-time relationship becomes nearly linear, suggesting partial disruption of the montmorillonite double-layer structure.

The expansion deformation behavior of geopolymer-treated montmorillonite was analyzed using the primary swelling coefficient (C_{ps}) [1,2], which is defined as:

$$C_{ps} = \frac{\Delta \varepsilon_p}{\Delta \log_{10}^{t}} \qquad \text{(S.1)}$$

Where: $\Delta\varepsilon_p$ represents the expansion strain during the main expansion stage (%); $\Delta \log_{10}^{t}$ represents the change in time (min).

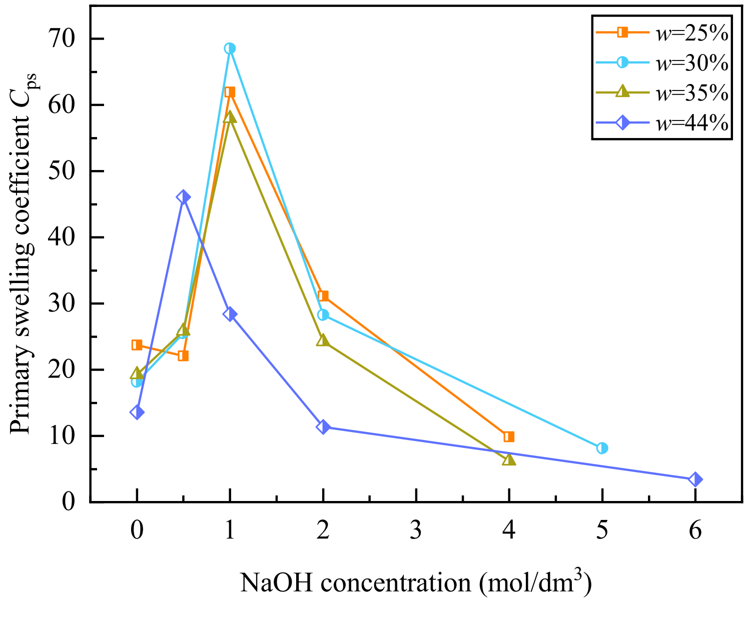

Figure S5. Primary swelling coefficient as a function of NaOH concentration at varying initial water contents

The time interval distinguishing the primary and secondary swelling stages was determined based on the findings of Yuan [2] and Ye[1], as illustrated in Figure S5. The variation of the primary swelling coefficient with NaOH concentration follows a

pattern similar to that of the overall free swell ratio (FSR)—initially increasing with higher alkaline concentration and subsequently decreasing.

S5. Formation of Sodium Hydroxide Crystals on the Porous Stone

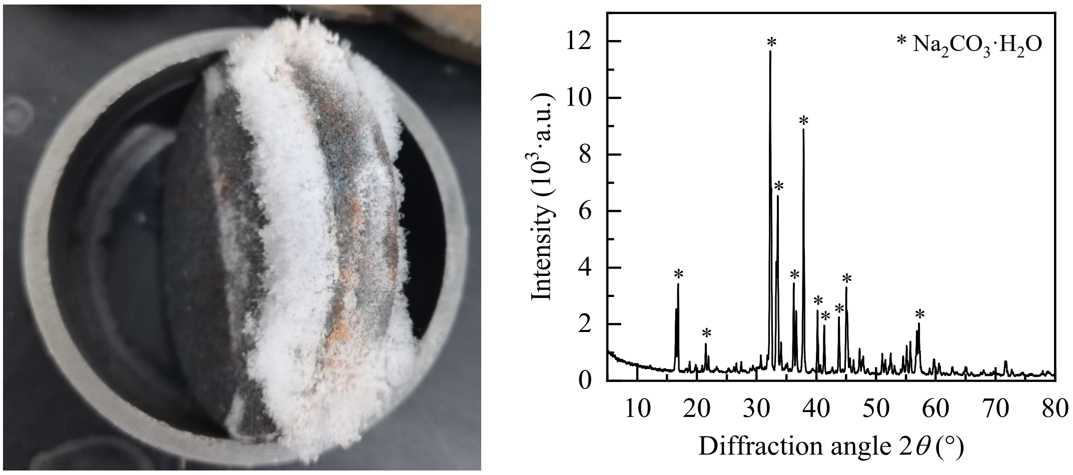

(a) (b)

Figure S6. Crystallized sodium carbonate on the surface of permeable stone and corresponding XRD pattern. (a) Crystallized sodium; (b) XRD pattern.

During the swelling test of the geopolymer-treated soil with an NaOH concentration of 6.00 mol/dm^3, a white powdery substance was observed on the porous stone, as shown in Figure S6a. XRD analysis identified this substance as $Na_2CO_3 \cdot H_2O$ (Figure S6b), indicating that an excess of Na^+ remained in the solution and subsequently reacted with atmospheric CO_2 to form sodium carbonate. In contrast, no sodium carbonate was detected on the porous stones of specimens treated with 2.00 mol/dm^3 NaOH or other lower concentrations. This further suggests that at 6.00 mol/dm^3, the calcium-based montmorillonite had been completely converted into sodium-based montmorillonite.

S6. Effect of NaOH Concentration on the Mechanical Strength of Modified Bentonite

To investigate the effect of NaOH concentration on the mechanical behavior of geopolymer-treated bentonite, specimens were prepared with initial water contents of

w = 35% and 44% and a fixed fly ash content of FA = 9%. NaOH solutions of varying concentrations were used as the alkaline activator. All specimens were compacted to an initial void ratio of e = 1.1. After preparation, the specimens were demolded, sealed to prevent moisture loss, and cured for 1 day under controlled conditions at a temperature of 22 ± 2 °C and a relative humidity of 70 ± 2%. Unconfined compression tests were then conducted to evaluate the strength characteristics of the treated bentonite, The results are shown in Figure S7.

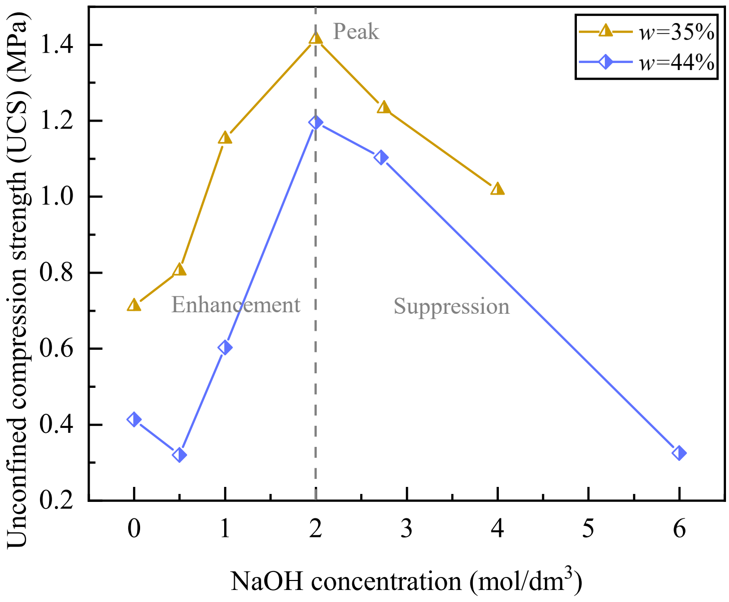

Figure S7. Evolution of unconfined compression strength (UCS) with NaOH concentration for geopolymer treated bentonite under different initial water contents.

Figure S7 illustrates the variation in unconfined compressive strength (UCS) of geopolymer-treated bentonite as a function of NaOH concentration for two different initial water contents (35% and 44%). For both water contents, the UCS initially increases with increasing NaOH concentration, reaches a peak at approximately 2 mol/dm^3, and then decreases with further increases in concentration.

At an initial water content of 35%, the strength increases from approximately 0.72 MPa at 0 mol/dm^3 to a peak value of about 1.41 MPa at 2 mol/dm^3, followed by a gradual decline to approximately 1.02 MPa at 4 mol/dm^3. A similar trend is observed

at an initial water content of 44%, although the overall strength values are lower. Specifically, the strength increases from approximately 0.41 MPa at 0 mol/dm^3 to a peak of about 1.20 MPa at 2 mol/dm^3, and then decreases significantly to approximately 0.33 MPa at 6 mol/dm^3.

In addition, at all NaOH concentrations considered, specimens with a lower initial water content (35%) consistently exhibit higher UCS than those with a higher initial water content (44%). This indicates that both NaOH concentration and initial water content play important roles in governing the strength development of geopolymer-treated bentonite.

S7. Repeatability of the Free Swell Ratio (FSR) Test

To evaluate the repeatability and reliability of the free swell ratio (FSR) measurements, parallel tests were conducted on three independently prepared specimens under identical conditions. The specimens were prepared with an initial void ratio of e = 1.1, NaOH concentration of c = 1.00 mol/dm³, initial water content of w = 25%, and fly ash content of FA = 9%. All specimens were prepared and tested in accordance with the standardized procedure described in Section 2.3 of the main text.

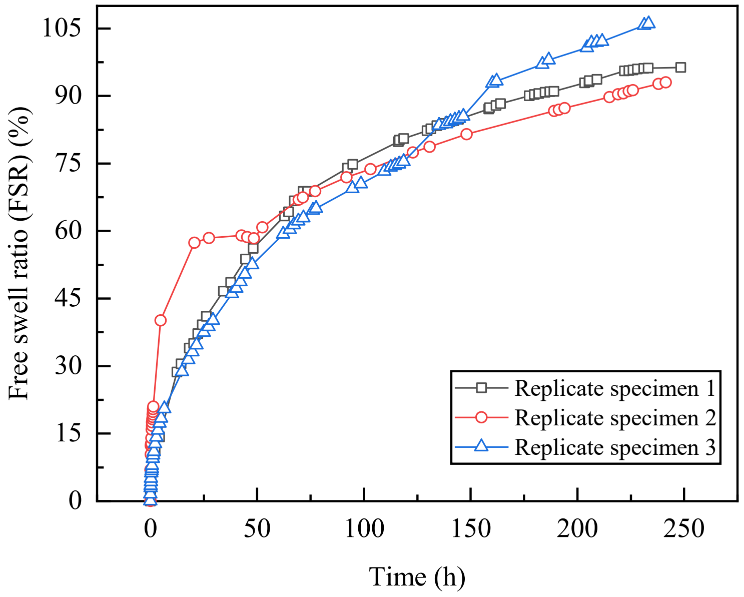

Figure S8. Time-dependent swelling curves of parallel Free Swell Ratio (FSR) specimens.

The time-dependent swelling curves of the three replicate specimens are presented in Figure S8. All specimens exhibited a consistent swelling evolution, characterized by a rapid initial increase followed by a gradual approach to equilibrium. The swelling curves showed close agreement throughout the test duration, and the equilibrium FSR values were comparable among the replicate specimens, with only minor deviations observed during intermediate stages.

Overall, the good agreement in both swelling trends and equilibrium values demonstrates the repeatability and stability of the FSR measurements. This consistency confirms that the specimen preparation method and testing protocol described in Section 2.3 of the main text provide reliable and reproducible results.

Reference

1. Ye, J. Effect of Montmorillonite Content and Sodium Chloride Solution on the Residual Swelling and Soil Water Characteristics of an Expansive Clay. PhD thesis, Wuhan University: Wuhan, 2019.
2. Yuan, S.; Liu, X.; Sloan, S.W.; Buzzi, O.P. Multi-Scale Characterization of Swelling Behaviour of Compacted Maryland Clay. *Acta Geotech.* **2016**, *11*, 789–804, doi:10.1007/s11440-016-0457-5.
